# A glimpse into the application of the immunomodulatory effect of IL-2 in systemic lupus erythematosus

**DOI:** 10.3389/fmed.2025.1552473

**Published:** 2025-04-23

**Authors:** Xin Xia, Rui Qu

**Affiliations:** ^1^School of Medicine, Jiangsu University, Zhenjiang, China; ^2^Faculty of Civil Engineering and Mechanics, Jiangsu University, Zhenjiang, China

**Keywords:** interleukin-2, SLE, immunotherapy, engineered T cells, IL-2

## Abstract

Systemic lupus erythematosus (SLE) is a systemic autoimmune disease, which is mainly caused by the imbalance of immune cells. Current treatment regimens predominately rely on corticosteroids and immunosuppressive agents, accompanied by various side effects. Interleukin-2 (IL-2) is deemed an important cytokine for innate immune cells and adaptive immune cells, especially for the promotion of Treg cells. By combining IL-2/IL-2R system with engineered T cell-based immunotherapies to enhance the therapeutic efficacy of engineered T cells shows its potential in autoimmune diseases. But the pleiotropy of IL-2 may cause simultaneous stimulation and systemic toxicity, limiting its therapeutic use. There is a growing focus on using IL-2 in combination strategies for synergistic immune enhancement. In this article, we review the IL-2/IL-2R signaling, including IL-2 dependent signaling and IL-2 independent signaling, and discuss its functions in regulation of different immune cells. In addition, we summarize major clinical application of low-dose IL-2 treatment in SLE with or without other agents, such as rapamycin, tocilizumab and rituximab, present the IL-2 variants and fusion proteins designed for SLE, and highlight the future trends for research on these cytokine-based immunotherapies. It will help to design further optimized IL-2-based therapy for SLE.

## 1 Introduction

Systemic lupus erythematosus (SLE) is a systemic autoimmune disease characterized by red speckles on the skin and multiple organ damage. The pathophysiology of SLE is complex that remains incompletely understood. Studies showed that the decreases of regulatory T (Treg) cells as well as the imbalance of effector T (Teff) cell subsets and Treg cell subsets may contribute to the pathogenesis of SLE. Current treatment regimens mainly rely on corticosteroids and immunosuppressive agents. These traditional treatments are likely to result in substantial side effects, such as various infections. Some cases thus become refractory or severe SLE. Sustained remission is achieved in only a small fraction of patients. So it is urgent to develop an effective treatment for SLE.

Interleukin-2 (IL-2) is considered as an important cytokine for the inflammatory immune responses against pathogens and tumors. It was previously introduced in a high dose setting to treat malignant diseases. During 1980s, the clinical application of high-dose IL-2 treatment showed the regression of metastatic renal carcinomas and melanomas (1). In 1992, the therapeutic application of IL-2 (Aldesleukin, trade name Proleukin) in treating the renal cancer was approved by the US Food and Drug Administration (FDA) (2, 3). It was subsequently approved for patients with metastatic melanoma in 1998. Later studies show that IL-2 is key to immune tolerance (4). It is essential to the differentiation and proliferation of Treg cells. Currently, clinical studies have shown the safety and efficacy of low-dose IL-2 in the treatment of SLE (5). Compared to the traditional treatment, low-dose IL-2 treatment is effective without increased infection incidence. However, the optimal dosage of IL-2 therapy remains to be determined. This review takes combination therapies into consideration. By comparing the curative effect of using IL-2 as single agent or as a component of combination therapies to further explore the optimized clinical application of IL-2 in SLE treatment, such as drug dosage adjustment, selection of combination drugs, and patient management.

## 2 IL-2/IL-2R signaling

### 2.1 IL-2 dependent IL-2R signaling

**FIGURE 1:**
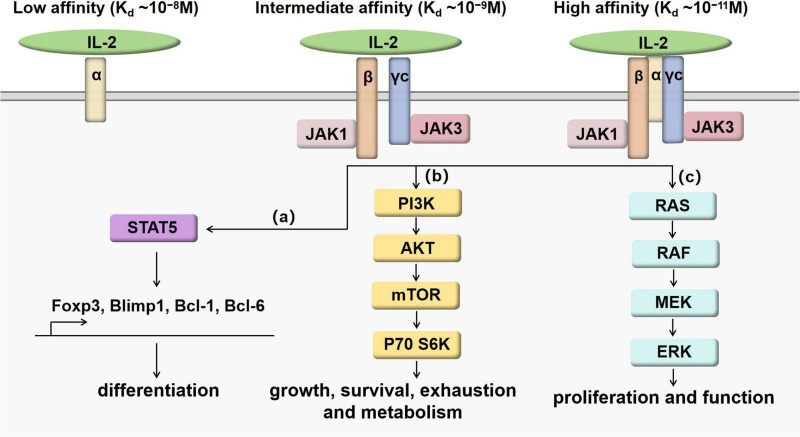
IL-2/IL-2R signaling pathways. When IL-2 binds with intermediate-affinity IL-2R or high-affinity IL-2R, the formation of cytokine-receptor complex leads to the activation of JAK1 next to IL-2Rβ and JAK3 next to γc. Although the binding affinity can be increased by IL-2Rα, IL-2Rα does not work as the signaling subunit. Downstream signaling pathways are presented as **(a–c)**. **(a)** IL- 2 promotes the phosphorylation of STAT5 affecting the expression of Foxp3, Blimp1, Bcl-1 and Bcl-6 to regulate the differentiation and maturation of T cell subsets. **(b)** IL- 2 promotes the activation of PI3K-AKT-mTOR pathway to regulate T cell growth, survival, exhaustion and metabolism. **(c)** IL- 2 promotes the activation of MAPK pathways to regulate T cell proliferation and function.

IL-2 is a 4-bundle α-helical protein of 15 kDa, produced by antigen-activated CD4^+^ T cells through the T cell receptor (TCR) and CD28 co-stimulation. CD8^+^ T cells, natural killer (NK) cells, natural killer T (NKT) cells and dendritic cells (DCs) also secrete IL-2, but in lower quantities. IL-2 regulates immune responses via binding to IL-2 receptor (IL-2R). IL-2R has three subunits, including IL-2Rα (CD25), IL-2Rβ (CD122) and γc (CD132), which can form high-affinity, intermediate-affinity and low-affinity IL-2R by different combinations (6). High-affinity IL-2R (K_*d*_∼10^–11^M) has three subunits IL-2Rα, IL-2Rβ and γc. Intermediate-affinity IL-2R (K_*d*_ ∼10^–9^M) comprises of two subunits IL-2Rβ and γc. Low-affinity IL-2R (K_*d*_ ∼10^–8^M) contains only IL-2Rα. When IL-2Rα and IL-2Rβ are co-expressed without γc, there is “pseudo-high affinity” binding (K_*d*_ ∼ 10^–10^M), but no signaling occurs (7, 8). The functional receptors are intermediate-affinity IL-2R and high-affinity IL-2R. Intermediate-affinity receptors are expressed by resting T and NK cells, whereas high-affinity receptors are present on activated lymphocytes (7). After IL-2 bind to intermediate-affinity IL-2R, high-affinity receptors are formed by the rapid induction of IL-2Rα, increasing responsiveness to IL-2. Major downstream signaling pathways of IL-2 receptor include STAT5, PI3K and MAPK pathways (9), as shown in Figure 1. The combination of IL-2 and IL-2R can recruit JAK1 and JAK3 to the receptor complex, leading to the phosphorylation and activation of STAT5. Activated STAT5 can migrate to the nucleus and promote the transcription of vital genes such as Foxp3 (10), Blimp1 (11, 12) and Bcl-6 (11, 13) to regulate immune cell differentiation. After stimulated by IL-2, the PI3K-Akt pathway can facilitate cell growth and survival by suppressing exhaustion and increasing metabolism (14, 15). The MAPK pathway mainly plays an important role in the process of cell proliferation and function (16).

### 2.2 IL-2 independent IL-2R signaling

IL-15 belongs to the IL-2 cytokine family, mainly produced by DCs and monocytes/macrophages. IL-15R is a heterotrimeric receptor, including γc, IL-2Rβ and IL-15Rα. IL-15Rα is predominantly expressed on monocytes and DCs, which binds with IL-15 with high affinity (K_*d*_∼10−11 M). It can trigger intracellular signaling pathways following binding with IL-15 (17, 18). IL-15 shares many biological activities with IL-2, including the stimulation of activated T cell proliferation and the generation of cytotoxic effector T cells. Despite these similarities, IL-15 and IL-2 have distinct functions, IL-15 is required for the activation and maintenance of NK cells, the persistence of memory CD8^+^ T cell, the promotion of immunoglobulin synthesis by B cells, as well as the regulation of lymphoid homeostasis (19, 20). By contrast, IL-2 is the major force for the development and maintenance of Treg cells (21).

## 3 Regulation of immune cells

### 3.1 Regulation of innate immune cells

Activated monocytes and macrophages constitutively express IL-2Rβ, which is regulated by IL-2 and IFN-γ (22, 23). IL-2 can increase the cytotoxicity of macrophages and induce activated macrophage to be resistant to infection (24, 25). It is speculated that IL-2R can absorb IL-2 during inflammation to influence macrophage differentiation and restrain T cell expansion and function through this competitive binding (26). DCs can express all three subunits of IL-2R. IL-2 can stimulate DC formation and substantially expand type 1 DCs which improve anti-cancer immunity (27, 28). NK cells do not express IL-2Rα, but constitutively express IL-2Rβ. The majority of IL-2R presented on NK cell surface is low-affinity dimerized complex (27). It is found that IL-2 promotes the cytotoxicity of NK cells in a dose dependent manner (29). Innate lymphoid cells (ILCs) are a population of lymphocytes that distribute in all organs serving as frontline immune guard. IL-2 can efficiently promote the proliferation and survival of group 2 innate lymphoid cells (ILC2s) (30). IL-2 enhances the effector function of ILC2s by stimulating the cytokine production such as IL-5, IL-10 and IL-13 (27).

### 3.2 Regulation of adaptive immune cells

IL-2 can promote the differentiation of Th1, Th2, Th9, and Treg cells (31–36), but suppress the generation of Th17 (37, 38) and Tfh cells (39–41). During Th1 cell differentiation, IL-2 can induce IFN-γ production and increase expression of T-bet (42). Enhanced mTORC1 signaling by IL-2 can positively influence the differentiation of Th1 cells (40). IL-2 can control Th2 cell fate via the transcription factor STAT5 by regulating the Th2 cytokine gene cluster and stabilizing the accessibility of *Il4ra*, the gene encoding the IL-4 receptor α-chain (31–33). Th9 cell differentiation is controlled by the competition between IL-2 and IL-21, by influencing STAT5 binding site at the *Il9* gene. IL-2 can cut down the expression of Bcl-6, while the increased Bcl-6 expression can inhibit IL-9 production. IL-21 has opposing actions (43). IL-2 can stabilize Foxp3 expression to maintain lineage identity of Treg (34). It is required for the suppressive function and the thymic development of Treg (35, 36). IL-2 can inhibit CD4^+^ T cells from polarization into Th17 and Tfh cells by reciprocal actions of STAT3 and STAT5, partly contributing to enhanced differentiation of Th1 cells (37, 41). Intriguingly, after Th17 cells establish their identity, IL-2 will promote the proliferation of these cells (44). In addition, IL-2 is shown to inhibit germinal center (GC) formation. There are impaired influenza-specific GCs, long-lived IgG responses with IL-2 administration (39).

Apart from regulating the differentiation of CD4^+^T cells, IL-2 is also important for CD8^+^ T cells to regulate their effector function and memory formation, depending on their IL-2Rα expression. After viral infection, CD8^+^ T cells with high levels of IL-2Rα tend to become terminally differentiated, short-lived effector cells and express cytolytic molecules and cytokines, mediated by Blimp1 induction. While those with low levels of IL-2Rα prefer to increase Bcl-6, IL-7Rα and CD62L expression and preferentially turn to memory cells (41, 45). Besides, exhausted CD8^+^ T cells express higher levels of IL-2Rβ. Downregulation of IL-2Rβ leads to the reverse of exhausted phenotype, suggesting its the ability to rescue the exhausted state (46).

### 3.3 Regulation of engineered T cells

Adoptive transfer of engineered T cells has emerged as a potential therapeutic strategy for autoimmune diseases. These genetically modified autologous T cells were initially designed for hematological malignancies. Honaker et al. used homology-directed repair (HDR)-based gene editing to permit thymic T cells to express Foxp3 stably and robustly. These edited thymic Tregs can mediated suppressive activities in the models of inflammatory disease (47). Cook et Al. developed a gene-editing strategy to transform peripheral blood CD4+ T cells into Treg-like cells through the site-specific integration of a strong promoter upstream of the *Foxp3* gene. These edited cells also showed immuno-suppressive function (48). It is suggested that engineered Treg cells may exhibit therapeutic potential in a variety of autoimmune disorders (47, 49). However, the deficiency of appropriate cytokine support can lead to poor engraftment of the transferred T cells. It is reported that orthogonal IL-2 and IL-2Rβ system can increase the expansion of engineered T cells. This expansion is selective towards orthogonal IL-2Rβ expressing cells with no appreciable signaling on wild-type T cells. Orthogonal IL-2 can induce IL-2R signaling and support the proliferation of both an IL-2-dependent cell line and primary T cells transduced to express orthogonal IL-2Rβ. These data highlight the potential of combining an orthogonal cytokine approach with T cell-based immunotherapies to enhance the therapeutic efficacy of engineered T cells (50). Besides, the orthogonal IL-2/IL-2Rβ system may be able to eliminate the need for lympho-depleting chemotherapy that is usually used as preconditioning before engineered T cell therapy (51). These proofs showed the potential utility of IL-2/IL-2Rβ system for selective expansion of engineered T cells in autoimmune disorders (52).

### 4 High-dose or low-dose IL-2

Most clinical studies use the human recombinant IL-2 analog aldesleukin, which has a similar biological activity and a nearly identical biochemical structure compared to the native human IL-2 protein. High-dose IL-2 often uses over 100 million IU per day, while low-dose IL-2 therapy uses 0.3–3 million IU per day. High-dose IL-2 is an FDA approved drug for the treatment of metastatic melanoma and is associated with durable responses in a subset of patients, making it an option for the patients who do not respond to other therapeutic strategies (53). It is reported that high-dose IL-2 can make the stimulatory effects on Treg cells, which induce effector CD8^+^ T cell differentiation and increase the pool of self-reactive CD8^+^ T cells (54). A high dose of IL-2 therapy can cause Treg expansion in mice, which dampens immune responses against self-antigens including certain tumor antigens and renders mice susceptible to CD8^+^ T cell-mediated experimental autoimmune diabetes. In addition, high-dose IL-2 has considerable side effects such as vascular leak syndrome, and usually shows significant toxicity, which limit its efficacy. So high-dose IL-2 should be used with careful patient screening and treatment in malignant conditions, which is not justifiable and effective to applied in autoimmune diseases.

Low-dose IL-2 is more suitable for autoimmune diseases (55). Low-dose IL-2 therapy mainly activates Treg cells that express extensive level of high-affinity IL-2 receptor subunit IL-2Rα. It is reported that low-dose IL-2 treatment promotes Treg cells as well as inhibits Th17 cells and Tfh cells, accompanied by marked reduction of disease activity in patients with SLE (56). Moreover, low-dose IL-2 also sustains cellular immunity with enhanced NK cells (5). The benefit of low-dose IL-2 therapy is seen to be dependent on the expansion of immune tolerance-inducing Treg cells as well as the suppression of Teff cells, including Th17 cells and Tfh cells, contributing to the restoration of immune homeostasis and improvement of autoimmune inflammatory conditions.

## 5 Low-dose IL-2 and combination therapy in SLE

By searching the ClinicalTrials.gov database for the terms “IL-2” and “systemic lupus erythematosus,” we retrieved 18 clinical trials fulfilled our inclusion criteria. We summarize the available clinical results from early-stage to phase 3 clinical trials in Table 1. In order to avoid the side effects of IL-2, there are some engineered variants of IL-2-based therapeutics, such as IL-2 muteins and fusion proteins (57). Mutated forms of IL-2 are mainly receptor-biased IL-2, to make IL-2 prefer to bind to CD25 or CD122. CD25-biased IL-2 can selectively stimulate Treg cells, which is more suitable for SLE therapy. There are some approaches to selectively increase Treg cell expansion and induce the toxicity of IL-2, including PEGylated IL-2 and IL-2 fusion protein. PEGylated IL-2, including NKTR-358 (58, 59), STK-012 (60), and Dual-31/51-20K (61), is a mutated IL-2 which is permanently linked to PEG. It can obstruct the CD122 binding site and thus induce a CD25 bias. In addition, the linked PEG carrier protein can make IL-2 slowly released *in vivo*, which increases the half-life of IL-2. IL-2 fusion protein, including BAY 50-4798 (62), NHS-IL2 (63, 64), IL233 (65), AMG592 (66), and PT101 (67), is IL-2 mutein with reducing affinity for CD122. BAY 50-4798 has N88R mutation. NHS-IL2 is IL-2 coupled to anti-DNA-histone complex antibody. IL233 is formed by the fusion of the C-terminus (aa109-aa266) of IL-2 and IL-33 molecules. AMG592 is the fusion of IL-2 and Fc to enhance selectivity towards CD25 and extend the half-life of IL-2. PT101 is also Fc fused IL-2 mutein. Currently, NKTR-358, Dual-31/51-20K, AMG592, PT101 and IL233 are in the clinical trial of SLE.

**TABLE 1:** IL-2-based compounds for the intervention of SLE.

Clinicaltrials.gov identifier	Overview	Status	Intervention	Phase	Provider	References
NCT04077684	Efficacy and safety of low-dose IL-2 in patients with SLE: a multicenter, randomized, placebo-controlled trial	Recruiting (last update posted 2023-10-12)	Drug: IL-2	Phase 2	Zhanguo Li, Peking University People’s Hospital	
NCT05339217	Efficacy and immunological evaluation of telitacicept and low dose IL2 in the treatment of systemic lupus erythematosus	Recruiting (last update posted 2024-04-03)	Drug: telitacicept Drug: IL-2	Phase 3	Liu Tian, Peking University People’s Hospital	
NCT06255028	A study of CNTY-101 in participants with moderate to severe systemic lupus erythematosus (SLE)	Not yet recruiting (last update posted 2024-02-12)	Biological: CNTY-101 Biological: IL-2 Drug: lymphodepleting chemotherapy	Phase 1	Century Therapeutics, Inc.	
NCT02932137	Anti-infection of low-dose IL-2 in SLE	Completed (last update posted 2018-03-15)	Drug: IL-2	Not applicable	Zhanguo Li, Peking University People’s Hospital	(5, 86)
NCT02084238	Low-dose IL-2 (interleukin-2) treatment in SLE	Completed (last update posted 2020-04-03)	Drug: IL-2	Not applicable	Peking University People’s Hospital	(87)
NCT05262686	Efficacy and immunological evaluation of belimumab plus low dose IL-2 in the treatment of systemic lupus erythematosus	Unknown status (last update posted 2022-03-25)	Drug: belimumab Drug: IL-2	Phase 3	Peking University People’s Hospital	
NCT04397107	The therapeutic value and mechanism of recombinant human interleukin-2 on children with rheumatic diseases (SLE, pSS, JIA)	Completed (last update posted 2022-08-18)	Drug: IL-2	Not applicable	The First Hospital of Jilin University	(88, 89)
NCT02955615	ILT-101 in patients with active moderate to severe systemic lupus erythematosus (SLE) (LUPIL-2)	Completed (last update posted 2019-03-05)	Drug: ILT-101 Drug: Placebo	Phase 2	Iltoo Pharma	(90)
NCT05631717	The study of comparing the efficacy and safety of human umbilical cord MSCs and low-dose IL-2 in the treatment of LN	Recruiting (last update posted 2022-11-30)	Biological: human umbilical cord mesenchymal stem cells Drug: IL-2	Phase 3	The Affiliated Nanjing Drum Tower Hospital of Nanjing University Medical School	
NCT03312335	Low-dose interleukin-2 for treatment of systemic lupus erythematosus (Charact-IL-2)	Completed (last update posted 2020-08-19)	Drug: low-dose Aldesleukin (Proleukin^®^)	Phase 2	Onur Boyman, MD, University of Zurich	
NCT02465580	A pilot-study with low-dose hrIL-2 for the treatment of systemic lupus erythematosus	Unknown status (last update posted 2015-06-08)	Drug: hrIL-2 active Drug: hrIL-2 placebo	Phase 2	Zhanguo Li, Peking University People’s Hospital	
NCT04433585	A study of LY3471851 in adults with systemic lupus erythematosus (SLE) (ISLAND-SLE)	Completed (last update posted 2024-04-23)	Drug: LY3471851 Drug: placebo	Phase 2	Nektar Therapeutics	
NCT01988506	Induction of regulatory T cells by low dose IL-2 in autoimmune and inflammatory diseases (TRANSREG)	Completed (last update posted 2021-07-20)	Drug: IL-2	Phase 2	Assistance Publique - Hôpitaux de Paris	(88)
NCT05544448	*In vitro* effect study of interleukin-2 muteins on regulatory T cells of patients with different autoimmune, allo-immune or inflammatory diseases (MuTreg)	Not yet recruiting (last update posted 2022-09-16)	Other: blood sample taken at a single time point	Not applicable	Assistance Publique - Hôpitaux de Paris	
NCT04136106	The incidence of infection in treatment of low-dose IL-2 of SLE patients	Recruiting (last update posted 2019-10-23)	Drug: IL-2	Not applicable	Peking University People’s Hospital	
NCT03451422	Safety, tolerability, pharmacokinetics, pharmacodynamics, and immunogenicity of Efavaleukin Alfa in participants with systemic lupus erythematosus	Completed (last update posted 2023-07-09)	Drug: Efavaleukin Alfa Drug: placebo	Phase 1	Amgen	(86)
NCT04433585	A study of LY3471851 in adults with systemic lupus erythematosus (SLE) (ISLAND-SLE)	Completed (last update posted 2024-04-23)	Drug: LY3471851 Drug: placebo	Phase 2	Nektar Therapeutics	
NCT04987333	Study of Efavaleukin Alfa in healthy Chinese, Japanese, and Caucasian participants	Completed (last update posted 2024-03-29)	Drug: Efavaleukin alfa	Phase 1	Amgen	

### 5.1 Low-dose IL-2 alone

Compared to health controls, SLE patients have decreased Treg and Tfr cells as well as increased Tfh cells, while Th17 cells show no significantly difference between two groups, suggesting that Treg cells play a predominate role in the pathology of SLE. Active patients have a higher ratio of activated Tfh/Tfr cells compared to inactive patients. Furthermore, serum IL-2R level is associated with SLEDAI-2K, anti-dsDNA, C3, ferritin level, CRP, urine sediments and leukocyturia, suggesting its promising potential for SLE activity evaluation (68). After low-dose IL-2 therapy, there is a fourfold increase in circulating Treg cells, whereas Th17 cells are increased slightly in SLE patients (69). In refractory SLE, there are increased Th17 and Treg cells and decreased Th17/Treg ratio after IL-2 administration with markedly reduced clinical symptoms. The urine of discharged patients with lupus nephritis was negative after 3 weeks (70). The loci of *Foxp3* at STAT binding sites are marked by bivalent histone modifications. After low-dose IL-2 stimulation, STAT3 and STAT5 selectively bind on *Foxp3* and *Bcl-6* gene loci accompanied by suppressing H3K27me3, leading to the conversation of memory Tfh to functional Tfr cells and the decrease of plasmablasts (71, 72). It is suggested that low-dose IL-2 is conducive to the formation of immune tolerance, the recovery of immune function and the relieve of clinical symptoms without increasing side effects, worthy of clinical application.

### 5.2 Low-dose IL-2 combined with rapamycin

Rapamycin acting as a cell cycle inhibitor works on mTOR, which is a downstream target of the PI3K pathway. The use of rapamycin can inhibit the proliferation of contaminating conventional effector cells, facilitate the growth of Treg cells *in vitro* (73), and promote engraftment and survival of engineered Treg cells *in vivo* (48). Recent studies have shown that rapamycin is effective in reducing disease activity of SLE with limited side effects (74). Low- dose IL- 2 can selectively enhance Treg cells function and rapamycin can promote the proliferation of Treg cells, the combination of the low- dose IL- 2 and rapamycin has been considered to be a good pair of partner. It is reported that low- dose IL- 2 combined with rapamycin results in an increase in the absolute counts of Treg cells in refractory SLE patients, 26.2% patients with refractory SLE ware improved (75, 76). Addition of rapamycin to cultures containing IL-2 further increases the frequency and absolute number of functional Treg. This increase is not due to selective proliferation of Foxp3^+^ cells. Instead, it is resulted from an increase in the frequency of Foxp3^+^ cells induced in G0 phase through delayed activation, while the presence of IL-2 promotes survival and proliferation of the Foxp3^+^ population (77). Thus, low-dose IL-2 combined with rapamycin may provide a better curative effect for SLE patients by promoting induction, survival and expansion of functional Treg. Besides, the combination of low-dose IL-2 and rapamycin could reduce the dosage of prednison (76). It is beneficial for the SLE patients with serious adverse reactions to the use of corticosteroids.

### 5.3 Low-dose IL-2 combined with tocilizumab

Tocilizumab is a humanized IgG1 monoclonal antibody targeting IL-6. It is reported that two SLE patients with persistent high-grade fever regain normal body temperatures after treatment with tocilizumab, and other symptoms such as arthralgia gradually are relieved as well (78). Although the observed clinical responses are promising, neutropenia may limit the maximum dosage of tocilizumab in patients with SLE (79). It is necessary to explore the combination therapy of tocilizumab to improve its efficacy. It is found that low doses of IL-2 combined with tocilizumab are safe and effective in rheumatoid arthritis (RA) patients (80). The results of this study show that low-dose IL-2 combined with tocilizumab treatment can reduce arthritis symptoms in RA patients, promote the more stable proliferation of Tregs, and suppress the proliferation of Th2, Th17, and Th17/Treg caused by IL-2 treatment alone, suggesting its great value for patients with immune disorders mediated by high levels of Th2 and Th17. Besides, the CRP levels were greatly decreased after the treatment of low-dose IL-2 and tocilizumab, compared to using IL-2 alone. Thus, low-dose IL-2 accompanied with tocilizumab may be an alternative treatment for the patients in the active stage of SLE who fail to respond to initial antibiotics and high-dose glucocorticoids. It is also benefical for the patients who have low Treg cells along with high Th cell. Combined tocilizumab can effectively reverse reduced Treg cells and increased Th cells at the same time contributing to rebuild immune homeostasis.

### 5.4 Low-dose IL-2 combined with rituximab

Rituximab is a humanized monoclonal antibody targeting CD20 on B cells. The treatment of rituximab can greatly prevent B cells reproduction. The evidences show that rituximab is effective and safe in the treatment of SLE, especially refractory SLE (81, 82). It is indicated that rituximab can improve the symptoms of the SLE patients with neuropsychiatric involvement and reduce the oral steroid dose (83). A recent case report showed that IL-2 plus rituximab can rapidly relieve the symptoms of SLE, such as fever, rash and mucosal ulcer (84). Apart from rituximab, belimumab which also targets B cell has shown its efficacy in the treatment of SLE (85). Belimumab can inhibit the B cell activating factor (BAFF) to decrease the production of autoantibodies. Though there is no clinical trials using IL-2 accompanied with belimumab. It still provides a novel strategy for the IL-2 combination therapy.

The main features of the above therapies are summarized in Table 2.

**TABLE 2:** The comparison of IL-2 and its combination therapy.

	Patient characteristics	Administration	Immunological analysis	Efficacy	Safety
IL-2 alone	Sixty patients with active SLE were recruited, and divided into two groups: IL-2 and placebo.	IL-2 group received IL-2 (1 MIU/day) subcutaneously for 2 weeks, followed by a 2-week break as one treatment cycle.	There were significant expansions of Treg cells and NK cells	The complete remission rate was 55.17% at week 12 and 65.52% at week 24.	Injection-site reactions (33.1%) Transient influenza-like symptoms (10.3%) Transient fever (14.4%)
IL-2 with rapamycin	Fifty refractory SLE patients and 70 healthy controls were recruited.	Refractory SLE patients received low dose of IL-2 (100 WIU, 3-5 d/month, subcutaneous injection) and rapamycin (0.5 mg, once every other day, oral).	There were increased number of Tregs of at week 6, week 12 and week 24. The number of Th17 cells was unchanged.	After 6, 12 and 24 weeks of treatment, the SLEDAI score was significantly reduced.	A combination of low-dose IL-2 and rapamycin could significantly reduce the dosage of prednison.
IL-2 with tocilizumab	Fifty adults with active RA were recruited, and divided into three groups: control, IL-2, and IL-2+tocilizumab.	IL-2 group received IL-2 (0.5 MIU/day). IL-2+tocilizumab group received IL-2 (0.5 MIU/day) along with one dose of tocilizumab (8 mg/kg, maximum dose: 800 mg).	There was a violent proliferation of Treg cells in both IL-2, and IL-2+tocilizumab groups. The absolute number of Th1, Th2, and Th17 cells in the IL-2 with tocilizumab group showed a decreasing trend.	There were greatly reduced disease activity indicators in both IL-2 and IL-2+tocilizumab groups, especially CRP levels.	All patients did not have obvious adverse reactions, including allergy, fever and infusion reaction. ALT levels were slightly increased after IL-2 and tocilizumab treatment, but within the normal range.
IL-2 with rituximab	A 16-year-old female SLE patient with Purtscher-like retinopathy was included.	Rituximab (100 mg for the first time and 200 mg later) was given once a week for 4 weeks. IL-2 (subcutaneous injections of 1 MIU every other day for 2 weeks, followed by a 2-week break) was given for 2 weeks. Anti-VEGF antibody was injected intravitreally to right eye. Imipenem/cilastatin was applied for antibiotic therapy	Not mentioned.	Rituximab plus IL-2 induced rapid clinical remission of the active SLE with the sight recovering. No recurrence of Purtscher-like retinopathy was reported during 6-year follow-up.	No obvious adverse reactions were mentioned.

## 6 SLE therapy evaluation

To date, there is no universal agreement regarding the optimal tools to assess SLE. The assessment of patients with lupus usually relies on five domains: disease activity, chronic damage resulting from lupus activity or its treatment, unwanted side effects of drugs, health-related quality of life, and economic impact. The primary disease activity instruments are systemic lupus erythematosus disease activity index (SLEDAI), British Isles lupus assessment group index (BILAG), and physician global assessment (PGA) (91). Recently, Diogo Jesus et al. develop a new SLE disease activity score system (SLE-DAS) based on multivariate linear regression analysis with improved sensitivity to the changes in SLE disease activity and higher accuracy of damage prediction (92). As trials are conducted in different individuals with different outcome measures assessed at different time points and different dosing schemes with or without other agents. A conclusive statement on the IL-2 therapy is not possible. But we can assess the efficiency, safety and indication of IL-2 therapy according to the existing evidence.

The results show that compared to the placebo group, SLE patients with active disease improve rapidly and significantly with low-dose IL-2 treatment. Higher proportions of Treg cells, reduced SLEDAI scores and resolution of clinical features are observed, along with decreased serological activities such as reduced autoantibodies and increased serum complements. Different from immunosuppressants and biologics which often increased infection incidence, low-dose IL-2 treatment is effective in SLE without increased infection incidence (5, 93). A common and typical dermatological change at the site of injection is characterized by pain, redness and swelling. This injection site reaction occurred in 33.1% of patients with SLE. A common systemic effect is fever, occurring in 14.4% SLE patients treated with low-dose IL- 2. Transient influenza-like symptoms occurred in 10.3% of patients with SLE. Most of these side effects are mild and recover quickly after discontinuation. There are no serious adverse events in all these studies, including neuropsychiatric manifestations of SLE (NPSLE), herpeszoster and pneumonia (94).

The difficulty of immunotherapy is to find the patients who can benefit from immunotherapy. Selecting appropriate predictive biomarkers is a high priority to distinguish patients who are more likely to respond to IL-2 treatment. We can take advantage of hypothesis-generating data to support clinical trials involving single agents and combinations. Gao et al. develop a mathematical model to find an effective range of IL-2 dosage which is defined by the ratio of CD4^+^Foxp3^–^ T and CD4^+^CD25^+^Foxp3^+^ Treg cells. They find that SLE patients with lower Treg cells are more likely to benefit from IL-2 treatment (95, 96).

## 7 Conclusion and perspective

Low-dose IL-2 treatment has great potential as a novel strategy to rebuild the immune homestasis in individuals with SLE. An emerging number of evidence from the clinical trials demonstrated the profound safety and efficacy of low-dose IL-2 in controlling the disease flares in SLE patients. Even refractory symptoms showed improvement. In addition, IL-2 can be utilized with other agents, such as rapamycin, tocilizumab and rituximab, to design a more accurate and personalized treatment plan. However, there are still a few points to be noted. Firstly, current IL-2 treatment is not cell-specific, which may lead to side effects. So it is better to redirect the specificity of IL-2 toward adoptively transferred T cells based on orthogonal IL-2/IL-2Rβ system that can afford precise targeting of IL-2–dependent functions to a specific cell type of interest. For example, targeting cytotoxic CD8+ T and NK cells in anti-cancer treatment, but stimulating Tregs other than effector cells in the treatment of autoimmune diseases. Secondly, due to its short half-life (15–30 min), IL-2 requires frequent applications. The injection frequency is usually once a day, which will increase the pain of patients. It is necessary to modify IL-2 to extend its half-life or change the mode of administration. For example, IL-2 has a molecular weight of 15 kd, which can be absorbed through the skin or nose. It is possible to design transdermal or transmucosal absorption dosage form. Thirdly, future studies need to consider how to find appropriate predictive biomarkers to indicate patients who is susceptible to IL-2 treatment which will improve the efficacy of IL-2 therapy in clinical application. Lastly, it is advisable to conduct more large-scale, multi-center clinical trials to explore its optimal dose and long-term implications, and to study the possibility of combining IL-2 with other novel immunotherapy methods such as engineered T cells.
